# Testosterone-Dependent miR-26a-5p and let-7g-5p Act as Signaling Mediators to Regulate Sperm Apoptosis via Targeting *PTEN* and *PMAIP1*

**DOI:** 10.3390/ijms19041233

**Published:** 2018-04-18

**Authors:** Jideng Ma, Yu Fan, Jinwei Zhang, Siyuan Feng, Zihui Hu, Wanling Qiu, Keren Long, Long Jin, Qianzi Tang, Xun Wang, Qi Zhou, Yiren Gu, Weihang Xiao, Lingyan Liu, Xuewei Li, Mingzhou Li

**Affiliations:** 1 Farm Animal Genetic Resource Exploration and Innovation Key Laboratory of Sichuan Province, Sichuan Agricultural University, Chengdu 611130, China; jideng.ma@sicau.edu.cn (J.M.); fanxiao123yu@126.com (Y.F.); Jinweizhang50@163.com (J.Z.); siyuanfeng_bioinfo@163.com (S.F.); huzihui2018@163.com (Z.H.); qiuwanling2016@163.com (W.Q.); longkeren@163.com (K.L.); longjin8806@163.com (L.J.); wupie@163.com (Q.T.); xun_wang007@163.com (X.W.); weihang.xiao@sicau.edu.cn (W.X.); lingyan.liu@sicau.edu.cn (L.L.); xuewei.li@sicau.edu.cn (X.L.); 2 Chengdu Polytechnic, Chengdu 610041, China; Qizhou0205@126.com; 3 Animal Breeding and Genetics Key Laboratory of Sichuan Province, Pig Science Institute, Sichuan Animal Science Academy, Chengdu 610066, China; guyiren1128@163.com

**Keywords:** testosterone deficiency, sperm motility, apoptosis, miRNA, exosome

## Abstract

Recent evidence suggests that testosterone deficiency can dramatically decrease the quality of sperm. MicroRNAs (miRNAs) are conserved mediators of post-transcriptional gene regulation in eukaryotes. However, the systemic regulation and function of miRNAs in sperm quality decline induced by testosterone deficiency has not been investigated. Here, we found that the sperm apoptosis was significantly enhanced and the sperm motility was dramatically decreased in hemicastrated pigs. We then used small RNA sequencing to detect miRNA profiles of sperm from pigs with prepubertal hemicastration (HC) and compared them with control libraries. We identified 16 differentially expressed (DE) miRNAs between the sperm of prepubertal HC and control (CT) pigs. Functional enrichment analysis indicated that the target genes of these DE miRNAs were mainly enriched in apoptosis-related pathways including the p53, mitogen-activated protein kinase (MAPK), and mammalian target of rapamycin (mTOR) pathways. Furthermore, gain- and loss-of-function analyses demonstrated potential anti-apoptotic effects of the DE miRNAs miR-26a-5p and let-7g-5p on sperm cells. The luciferase reporter assay confirmed that *PTEN* and *PMAIP1* are targets of miR-26a-5p and let-7g-5p, respectively. Spearman’s correlation analysis revealed significantly positive correlations between the sperm and its corresponding seminal plasma exosomes regarding the miRNA expression levels. In conclusion, testosterone deficiency-induced changes in the miRNA components of seminal plasma exosomes secreted by the genital tract may partially elucidate sperm miRNAome alterations, which are further responsible for the decline of sperm motility.

## 1. Introduction

Mammalian testes have two main functions, spermatogenesis and androgen secretion. Spermatogenesis occurs in the seminiferous tubules of the postpubertal testis [1], while androgen formation and secretion take place in the interstitium of Leydig cells [2]. Testosterone is the primary and best recognized androgenic steroid hormone, which has multiple physiological effects in various target organs and plays a key role in testicular development and spermatogenesis [3]. Testosterone deficiency causes faulty development of reproductive organs and abnormal spermatogenesis and eventually leads to male reproductive disorders that are clinically termed hypogonadism [4]. Previous studies indicated that androgen receptor (AR) knockout, whether in Sertoli cells (S-AR^−/y^) [5] or total AR knockout (T-AR^−/y^) in mice [6], induces spermatogenesis arrest at the diplotene premeiotic stage, causing oligospermia and infertility. As an indispensable clinical testosterone deficiency model [7], prepubertal hemicastration can dramatically induce hypertrophy of the remaining testis through the rapid proliferation of Leydig cells and Sertoli cells [8,9]; moreover, it can decrease sperm counts and semen volume in male beagles [10], but enhances daily sperm production in the remaining testis of aged rats [11]. These results highlight the important role of testosterone in spermatogenesis and semen quality; however, the molecular mechanisms involved still remain poorly understood.

MicroRNAs (miRNAs) are non-coding RNAs ~22 nucleotides long that are derived from stem-loop precursors (pre-miRNAs) and regulate eukaryotic gene expression at the post-transcriptional level [12,13]. During the past decade, miRNAs were shown to be involved in various biological processes including development [14], cell growth [15], and differentiation [16]. Semen is a complex viscous fluid composed of spermatozoa and seminal plasma from seminiferous tubules, the epididymis, and accessory glands (including seminal vesicles, the prostate, and bulb urethral glands) [17]. Normozoospermic and asthenozoospermic men have different sperm miRNA expression profiles, suggesting that miRNAs affect sperm quality through several potential target genes [18]. miR-34c is highly expressed in mouse sperm, where it is critical for the first cleavage division [19]. Seminal plasma contains exosomes produced by the epididymis and accessory glands [20] and is characterized by high cholesterol and sphingomyelin content and a complex protein composition in humans, rats [21,22], rams [23], and boars [24]. Recent reports found that seminal exosomes in humans and mice contain small non-coding RNAs (miRNAs and tRNA-derived RNA fragments (tRFs)) with potential regulatory functions that aid fertilization and the intergenerational inheritance of an acquired metabolic disorder [25,26,27]. Thus, we hypothesized that miRNAs in sperm cells and seminal plasma exosomes play vital regulatory roles in maintaining the normal process of spermatogenesis and are responsible for testosterone deficiency-induced male reproductive disorders.

To test our hypothesis, we systematically and comprehensively compared the miRNA profiles of sperm cells between prepubertally hemicastrated Yorkshire boars (HC) and normal controls (CT). We found that differentially expressed (DE) miRNAs were mainly enriched in pathways involved in cell apoptosis, including the mammalian target of rapamycin (mTOR) and p53 signaling pathways. Furthermore, gain- and loss-of-function analyses and dual luciferase assays demonstrated that endogenous miR-26a-5p and let-7g-5p have potential anti-apoptotic and pro-survival functions in sperm cells through targeting *PTEN* and *PMAIP1* genes. Further comparisons revealed high similarities in miRNA profiles between seminal plasma exosomes and sperm cells, both in HC and CT groups. Our study revealed that miRNAs in sperm cells and seminal plasma exosomes have important functions in male reproduction, suggesting they could be used as potential treatment targets or novel diagnostic markers for male infertility.

## 2. Results and Discussion

### 2.1. Establishment of a Testosterone Deficiency Model in Yorkshire Boars

**Figure 1:**
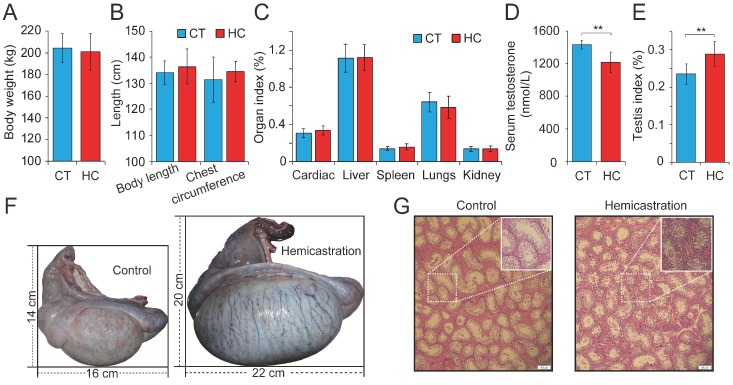

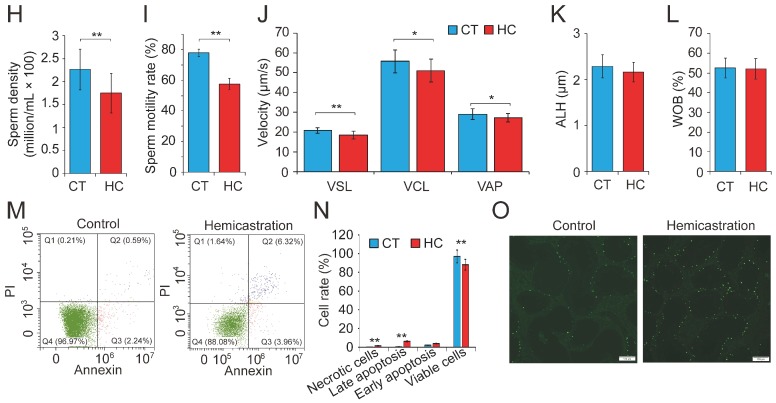
Altered profile of biological parameters between prepubertal hemicastration and control pigs. The influences of prepubertal hemicastration on porcine (**A**) body weight, (**B**) body size, and (**C**) main organ indexes. Prepubertal hemicastration significantly affected the (**D**) serum testosterone level, (**E**) testis index, and (**F**) testis size. (**G**) Histological analysis of the testis of control and hemicastrated pigs. Bars = 200 μm; 100× magnification; hematoxylin-eosin staining. (**H**) Sperm density of experimental pigs. (**I**) Sperm motility rate (%) represents the percentage of sperm showing motility. (**J**) Three motility parameters, including the curvilinear velocity (VCL, μm/s), average path velocity (VAP, μm/s) and straight line velocity (VSL, μm/s), were assessed for both experimental pig groups. (**K**) Amplitude of lateral head displacement (ALH, μm) for both experimental pig groups. (**L**) The wobble (WOB, %) measures the oscillation of the actual trajectory of sperm cells. (**M**,**N**) Sperm apoptosis rates of control and hemicastrated pigs were evaluated by flow cytometry analysis. (**O**) Apoptotic bodies in testicular tissues of control and hemicastrated pigs, detected as strongly green fluorescent cells. Bars = 100 μm; 200× magnification. CT and HC represent the prepubertally hemicastrated Yorkshire boars and normal controls, respectively. All data are expressed as means ± SD. * *p* < 0.05, ** *p* < 0.01.

Previous research has investigated testosterone deficiency by prepubertal hemicastration in mice, rats, beagle dogs, and boars from a physiological perspective, with a particular focus on endocrine levels. To explore the comprehensive effects of testosterone deficiency on the development of the main organs, we carried out hemicastration in prepubertal Yorkshire boars (seven days old) to establish a testosterone deficiency model. At the body maturation stage (10 months old), several phenotypic indexes were assessed in HC (*n* = 15) and CT (*n* = 15) pigs. This revealed no obvious alterations in body weight or length, chest circumference, or several organ indexes between HC and CT pigs (Figure 1A–C). However, significant differences were observed with respect to reproduction, including reduced serum testosterone (1197 ± 109 versus 1458 ± 37 nmol/L, *p* < 0.01), an increased testis index (0.24 ± 0.03 versus 0.28 ± 0.03%, *p* < 0.01), and an increased size of the remaining testis (length: 16.23 ± 1.82 versus 22.36 ± 2.19 cm, *p* < 0.01; width: 14.13 ± 1.55 versus 20.45 ± 1.97 cm, *p* < 0.01) in HC compared with CT pigs (Figure 1D–F). Histomorphological analysis showed that the hypertrophy of the remaining testis in hemicastrated pigs was associated with a significantly increased diameter of the seminiferous tubules (293.06 ± 45.25 μm, *n* = 5) when compared to CT pigs (261.03 ± 51.42 μm, *n* = 5) (*p* < 0.01) and obviously increased numbers of germ and Sertoli cells within the seminiferous epithelium (Figure 1G). These results were consistent with previous reports that prepubertal hemicastration was shown to induce hypertrophy of the remaining testis through the rapid proliferation of Leydig cells and Sertoli cells [8,9] and demonstrated the successful establishment of a testosterone deficiency model in Yorkshire boars.

Subsequently, 30 semen samples were collected from HC and CT pigs (*n* = 15 per group) for routine examination. Hemicastration was found to decrease sperm density (172 ± 46 million/mL in HC versus 225 ± 43 million/mL in CT, *p* < 0.01), weaken sperm motility (57.34 ± 3.87% in HC versus 78.52 ± 1.93% in CT, *p* < 0.01), and reduce three velocity parameters, including straight line velocity (VSL; 18.6 ± 2.3 μm/s in HC versus 21.4 ± 1.7 μm/s in CT, *p* < 0.01), curvilinear velocity (VCL; 51.7 ± 6.4 μm/s in HC versus 57.2 ± 5.9 μm/s in CT, *p* < 0.05), and average path velocity (VAP; 26.8 ± 2.6 μm/s in HC versus 28.3 ± 2.9 μm/s in CT, *p* < 0.05) (Figure 1H–J). No obvious differences were observed in the amplitude of lateral head displacement (ALH) or motility parameter wobble (WOB) between the two groups (Figure 1K,L). Flow cytometry analysis indicated that hemicastration significantly reduced sperm cell viability (88.34 ± 2.77% in HC versus 96.35 ± 2.65% in CT, *p* < 0.01) and induced apoptosis, which may be responsible for the reduced sperm density and motility (Figure 1M,N). Notably, extensive studies have indicated a significant negative correlation between sperm apoptosis and motility in vivo and in vitro [28,29,30]. These results indicated that testosterone deficiency by prepubertal hemicastration expectably reduced serum testosterone levels and induced a significant increase in sperm apoptosis and a dramatic decline in the sperm motility of Yorkshire boars. Moreover, the fluorescent terminal deoxynucleotidyl transferase-mediated dUTP nick end-labeling (TUNEL) assays showed that an obvious increase of cell apoptosis also occurred in the seminiferous epithelium of the remaining testis of the hemicastrated pigs (Figure 1O). Collectively, our results suggest that prepubertal hemicastration pigs compensate for the growth and development of their major organs at the expense of the normal development and physiological function of their remaining testis.

### 2.2. Testosterone Deficiency Dramatically Changes the Sperm miRNAome

**Figure 2:**
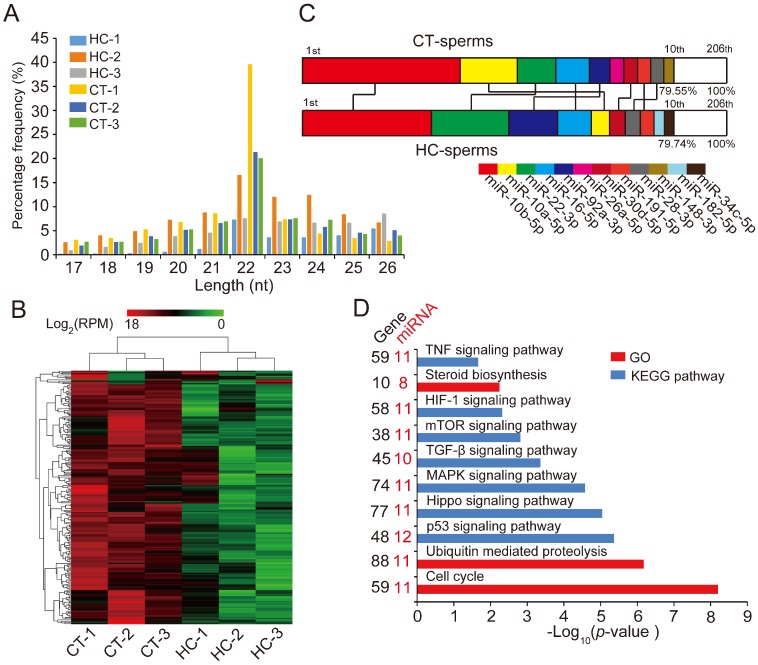

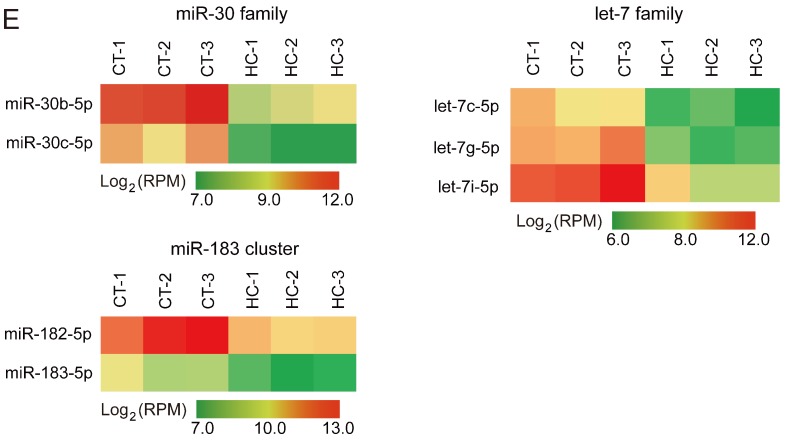
miRNA expression profiles of sperm cells in control and hemicastrated pigs. (**A**) The length distribution of identified miRNAs in all small RNA libraries. (**B**) Hierarchical clustering analysis for the expression of 206 known sperm miRNAs between control and hemicastrated pigs based on the Euclidean distance. Complete linkage hierarchic clustering was performed with the Euclidian distance measure. (**C**) Ranking analysis of the top 10 sperm miRNAs with the highest expression levels in control and hemicastrated pigs. The labels upper the bar represent the ranking of the top 10 miRNAs by expression, while the labels below the bar represent the accumulative % of the top 10 miRNAs in total read per million (RPM) of all expressed miRNAs. Seven miRNAs that are present in the top 10 position in both experimental groups are connected by lines. (**D**) Gene ontology (GO) and Kyoto Encyclopedia of Genes and Genomes (KEGG) pathway analysis of potential targets of the DE miRNAs. *p*-Values indicating the significance of enrichment were calculated by the Benjamini-corrected modified Fisher’s exact test. (**E**) Heat maps of the expression pattern of the DE sperm miRNA families and a cluster in control and hemicastrated groups. CT and HC represent prepubertally hemicastrated Yorkshire boars and controls, respectively.

Recent research showed that miRNAs play an important role in maintaining semen quality and ensuring normal fertilization [18,19]. To systematically investigate the mechanism of the specific decline of sperm quality in hemicastrated pigs at the miRNA transcriptome level, we isolated sperm cells from HC and CT semen samples (*n* = 3 per group), then extracted and sequenced small RNA using an Illumina HiSeq platform for miRNA profiling. We identified a total of 180 known pre-miRNAs encoding 206 mature miRNAs in six libraries (Table S1). The length distribution of identified miRNAs was consistent with the canonical size range of mammalian miRNAs [31] (Figure 2A), confirming the reliability of our small RNA-seq results.

Hierarchic clustering analysis for all 206 known miRNAs showed that HC and CT pigs were perfectly clustered into two independent branches based on the sperm miRNA transcriptome, which reflected the repeatability of our experimental treatment (Figure 2B). Ranking analysis showed that, although 80% (8/10) of the top 10 high-expressed miRNAs were shared between HC and CT sperm libraries, they had different expression levels (Figure 2C). These results demonstrated that prepubertal hemicastration markedly altered the sperm miRNA expression profile, which may further result in the disruption of sperm motility. Furthermore, 16 DE miRNAs were identified in HC versus CT sperm (defined as those exhibiting a Benjamini-corrected *p*-value ≤ 0.05 and a fold-change >2 or <0.5 in HC versus CT sperm) (Table S2). Subsequently, we used DIANA online software [32] for functional annotation of the sperm DE miRNAs. This revealed that the target genes of the DE miRNAs were mainly associated with the hypoxia-inducible factor-1 (HIF-1) signaling pathway, the cell cycle, and pathways involved in cell apoptosis, including the mTOR and p53 signaling pathways (Figure 2D). Notably, more than half of the sperm DE miRNAs (12/16, 75%) were enriched in the p53 signaling pathway; these DE miRNAs directly target important mRNA transcripts that coordinate apoptosis and cell survival. Additionally, among the 16 DE miRNAs, we detected two miRNA families (miR-30 and let-7 families) and one miRNA cluster (miR-183 cluster) whose members exhibited highly similar downregulated expression patterns in HC versus CT sperm (Figure 2E). These results indicated the potentially similar effects of these miRNAs on sperm quality and male fertility. Although the members of the let-7 miRNA family have been recognized as efficient tumor suppressors [33,34], they also exhibited fertility-related functions in recent studies, such as the upregulation of let-7 members in porcine sperm with abnormal morphology and motility [35] and the similarly high abundance of let-7 members in the ovaries and testes of *Portunus trituberculatus*, indicating their essential roles in gonadal development and function [36]. Moreover, deregulated sperm miR-182 was reported to mediate the paternal heredity of diet-induced obesity and metabolic disorders in mice [37]. Taken together, these results suggest that these sperm DE miRNAs play a vital function in sperm survival and maintaining sperm quality.

### 2.3. Overexpression of miR-26a-5p and let-7g-5p Improves Sperm Quality

**Figure 3:**
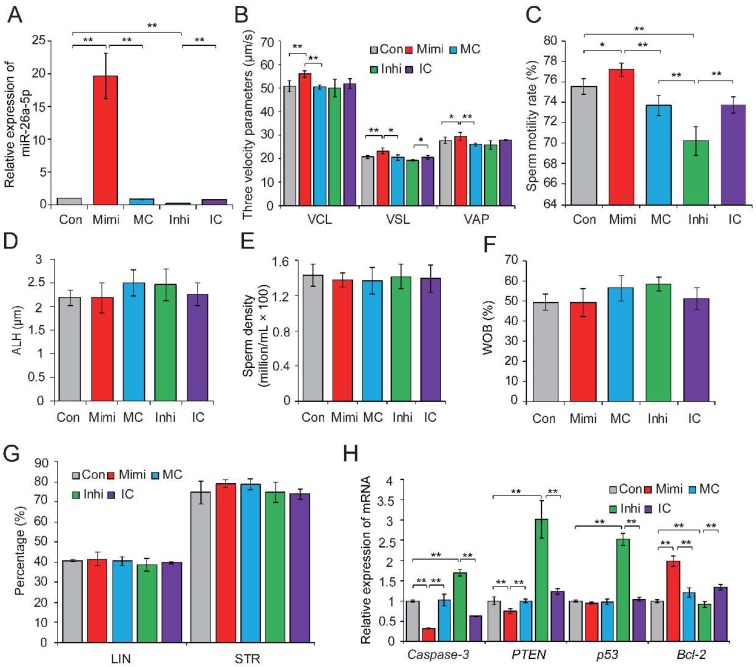
The miR-26a-5p-mediated regulation of sperm quality. (**A**) Relative expression levels of miR-26a-5p in control, mimic- and inhibitor-transfected sperm. (**B**) Effect of miR-26a-5p mimic and inhibitor on three velocity parameters of sperm cells, including curvilinear velocity (VCL, μm/s), average path velocity (VAP, μm/s) and straight line velocity (VSL, μm/s). (**C**) Effect of miR-26a-5p mimics and inhibitor on sperm motility rate (%). No significant changes were observed in (**D**) amplitude of lateral head displacement (ALH, μm), (**E**) sperm density (million/mL), (**F**) motility parameter wobble (WOB, %), or (**G**) linearity (LIN, %) and STR (%). (**H**) Effect of miR-26a-5p mimics and inhibitor on the expression levels of apoptosis-related genes. Con, control; Mimi, mimics; MC, mimic control; Inhi, inhibitor; IC, inhibitor control. CT and HC represent prepubertally hemicastrated Yorkshire boars and normal controls, respectively. Three independent experiments were performed in triplicate and all data are expressed as means ± SD. * *p* < 0.05, ** *p* < 0.01.

To further investigate the biological effects of DE sperm miRNAs on sperm quality, we selected miR-26a-5p and let-7g-5p, which were significantly downregulated in HC sperm cells and predicted to participate in apoptosis, for functional verification. Previous studies showed that miR-26a-5p protects RGC-5 cells against H_2_O_2_-induced apoptosis [38], while let-7g-5p inhibits oxidized low-density lipoprotein (ox-LDL)-induced apoptosis in endothelial cells by targeting caspase-3 [39]. Gain- and loss-of-function assays were performed by transfection of the miRNA mimic (or mimic control), or inhibitor (or inhibitor control) in freshly collected semen samples. qRT-PCR results confirmed the effective overexpression and downregulation of miR-26a-5p in sperm transfected with the miRNA mimic and inhibitor, respectively (*p* < 0.01) (Figure 3A). Phenotypic parameters of sperm evaluated 8 h after transfection showed that miR-26a-5p significantly improved sperm quality, by augmenting VCL (*p* < 0.01), VSL (*p* < 0.05), and VAP (*p* < 0.05) (Figure 3B) and enhancing sperm motility (*p* < 0.05) (Figure 3C). No significant changes were observed in ALH, sperm density, WOB, linearity (LIN), or straightness (STR) (Figure 3D–G). Moreover, overexpression of miR-26a-5p significantly reduced the expression of its predicted target gene *PTEN* and two other apoptosis marker genes (*caspase-3* and *p53*); whereas, it enhanced expression of the anti-apoptotic gene *Bcl-2* (*p* < 0.01) (Figure 3H). Similarly, the overexpression of let-7g-5p reduced sperm apoptosis and improved sperm motility (Figure S1A–H). These results highlighted the crucial role of miR-26a-5p and let-7g-5p in inhibiting sperm apoptosis and improving sperm motility.

### 2.4. PTEN and PMAIP1 Are Targets of miR-26a-5p and let-7g-5p, Respectively

To explore the mechanism by which miR-26a-5p and let-7g-5p significantly improved the reduction of sperm quality, we used TargetScan [40] and RNAhybrid [41] analyses to predict *PTEN* and *PMAIP1* as potential targets of miR-26a-5p and let-7i-5p. In the above gain- and loss-of-function assays, we found that both *PTEN* and *PMAIP1* mRNA levels were significantly reduced when miR-26a-5p or let-7g-5p were overexpressed in sperm (Figure 3H and Figure S1D).

**Figure 4:**
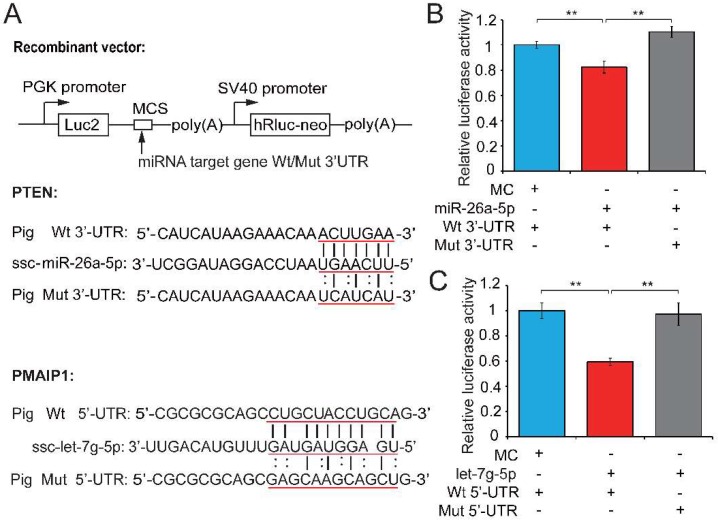
Testosterone-dependent miRNAs exert a pro-viability function by inhibiting pro-apoptotic factors. (**A**) Potential binding sites predicted by TargetScan [40] and RNAhybrid [41] for miR-26a-5p and let-7g-5p in the 3′-UTR of *PTEN* and the 5′-UTR of *PMAIP1*, respectively, and the mutant *PTEN* 3′-UTR and *PMAIP1* 5′-UTR used in our study. A luciferase reporter assay was performed by co-transfecting luciferase reporter containing the 3′-UTR of *PTEN* and 5′-UTR of *PMAIP1* (wild-type (Wt) or mutant (Mut)) with the mimic or control of miR-26a-5p and let-7g-5p into PK15 cells. The red underlined bases highlighted the miRNA seed sequences and their corresponding target sites in the mRNA UTR sequences. Luciferase activity was determined 48 h after transfection for (**B**) miR-26a-5p and (**C**) let-7g-5p. MC, mimics control. Three independent experiments were performed in triplicate and all data are expressed as means ± SD. ** *p* < 0.01.

We next performed a dual luciferase assay to demonstrate the relationships between candidate miRNAs and their potential target genes. The dual luciferase recombinant plasmid (pmirGLO-mRNA) constructed for *PTEN* and *PMAIP1* and mRNA and miRNA binding sites are shown in Figure 4A. Recombinant plasmid (with wild-type (Wt) or mutant (Mut) *PTEN* 3′-untranslated regions (UTR)) was co-transfected with a miR-26a-5p mimic into PK15 cells and the luciferase activity assay was performed 48 h after transfection. The upregulation of miR-26a-5p significantly repressed luciferase activity of the *PTEN* 3′-UTR reporter (0.82-fold change, *p* = 0.005; Figure 4B), whereas this repression was markedly abolished when the miR-26a-5p binding site in *PTEN* was mutated. The co-transfection of Wt or Mut *PMAIP1* 5′-UTR and let-7g-5p mimics into PK15 cells showed that let-7g-5p weakened the luciferase activity of the Wt *PMAIP1* 5′-UTR reporter (0.595-fold change, *p* < 0.001; Figure 4C). These results suggested that *PTEN* and *PMAIP1* are the respective target genes of miR-26a-5p and let-7g-5p. MiR-26a-5p appears to directly target *PTEN* and inhibit sperm apoptosis, while let-7g-5p reduced sperm apoptosis by targeting *PMAIP1*, then enhanced sperm motility and improved sperm quality. Our results also revealed that let-7g-5p binds *PMAIP1* 5′-UTR and exerts an inhibitor function. This is in keeping with previous reports that target mRNAs can be repressed as efficiently by miRNA binding sites in the 5′-UTR as in the 3′-UTR [42,43].

PTEN (also known as MMAC-1) is a multifunctional phosphatase whose lipid phosphatase activity induces apoptosis and cell cycle arrest through phosphoinositol-3-kinase/Akt-dependent pathway [44]. Recent studies have shown that PTEN can also exert its pro-apoptotic function through a mitochondria-dependent pathway [45,46,47], in which the PTEN–Bax complex was translocated from the cytosol to the mitochondria, triggering the dysfunction of mitochondrial membrane and downstream apoptotic processes, including the release of several pro-apoptotic factors (e.g., cytochrome c and ROS) and the activation of caspases (Caspase-3 and 9). PMAIP1, phorbol-12-myristate-13-acetate-induced protein 1 (also known as Noxa), is another pro-apoptotic factor [48]. It is a BH3-containing mitochondrial protein that is induced by p53 and contributes to p53-induced apoptosis by disrupting mitochondrial outer membrane integrity [49]. These indicate that both PTEN and PMAIP1 are implicated in the dysfunction of mitochondria and subsequent cell apoptosis, which will further affect the energy (ATP) production and sperm motility. Collectively, miR-26a-5p and let-7g-5p may indeed affect the sperm apoptosis and motility by targeting *PTEN* and *PMAIP1*, respectively.

### 2.5. Significant Correlation between Sperm and Seminal Plasma Exosomes Regarding miRNA Expression

**Figure 5:**
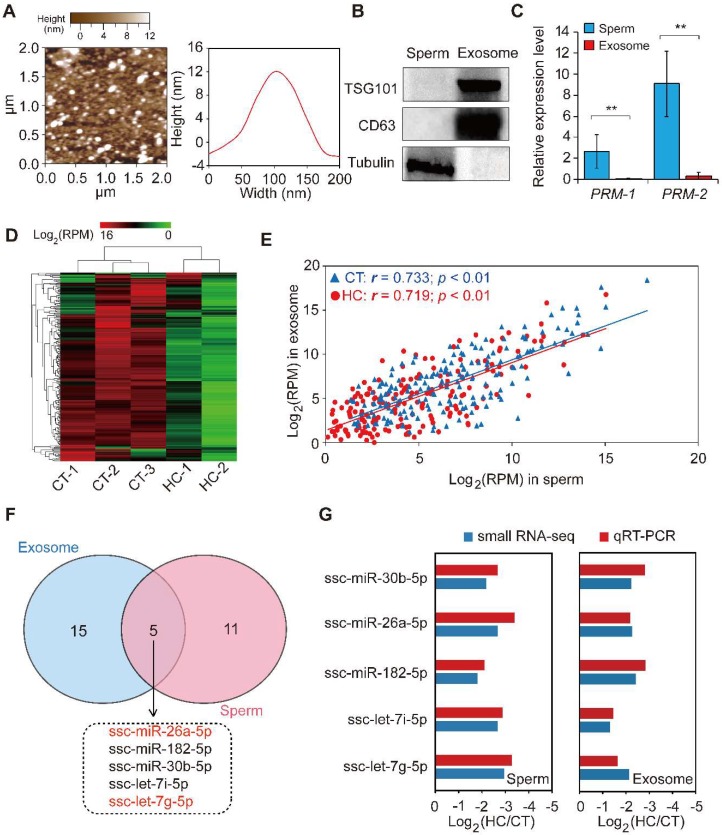
The comparison of miRNA data of sperm and seminal plasma exosomes. (**A**) Particle size distribution of CT-1 seminal plasma exosomes detected using atomic force microscopy. (**B**) Western blot analysis showing the enrichment of CD63 and CD81 and the absence of tubulin in CT-1 seminal plasma exosomes compared with sperm cell lysates. (**C**) The expression pattern of a sperm-specific marker, *PRM-1/2*, in sperm and seminal plasma exosomes. (**D**) Hierarchical clustering analysis for the expression of known exosomal miRNAs between control and hemicastrated pigs based on the Euclidean distance. (**E**) Spearman’s correlation of miRNA expression profiles between sperm and corresponding seminal plasma exosomes. (**F**) Venn diagram of DE miRNAs between sperm and seminal plasma exosomes in the HC versus CT group. (**G**) qRT-PCR validation of expression changes of five overlapping DE miRNAs between HC and CT groups. Three independent qRT-PCR experiments were performed in triplicate. All data are expressed as means ± SD. ** *p* < 0.01.

Exosomes are nano-sized vesicles secreted by various cell types. They function as intercellular signaling agents by transferring their contents, which include miRNAs, mRNAs, lncRNAs, proteins, and lipids [50]. Previous reports have suggested that seminal plasma contains nano-sized vesicles such as prostasomes and epididymosomes. To explore the miRNA expression pattern between sperm and corresponding seminal plasma exosomes, we isolated exosomes from seminal plasma by ultracentrifugation and used atomic force microscopy to investigate them at the nanometer scale. The results indicate that porcine seminal plasma contains a large number of membranous vesicles with an approximate width of 110 nm and a height of 12 nm (Figure 5A), which exhibited a canonical morphological feature consisting of the exosomes derived from other bodily fluids (i.e., saliva and urine) and cell culture fluid [51,52,53]. Western blot analysis showed that the exosome-specific markers CD63 and TSG101 were specifically enriched, while the cellular specific marker tubulin was absent in the seminal plasma exosome-like vesicles, when compared to sperm cell lysates (Figure 5B). This result further confirmed the real identity of exosomes isolated from porcine seminal plasma. Moreover, the sperm-specific marker *PRM-1/2* was shown by qRT-PCR to be highly expressed in sperm cells compared with seminal plasma exosomes (Figure 5C). These results demonstrate the presence of exosomes in seminal plasma, in keeping with the characteristics of typical exosomes listed by the International Society for Extracellular Vesicles [54]. Subsequent small RNA-seq using total RNA isolated from seminal plasma exosomes revealed extremely similar changes in the miRNA transcriptome of seminal plasma exosomes between CT and HC groups compared with sperm (Figure 5D, Table S3).

We further analyzed the Spearman’s correlation of the miRNA expression profile between sperm and corresponding seminal plasma exosomes, which was found to be significantly positive (CT: *r* = 0.733, *p* < 0.01; HC: *r* = 0.719, *p* < 0.01) (Figure 5E). Furthermore, 20 DE miRNAs were identified in seminal plasma exosomes between CT and HC groups (Table S4), of which five (including miR-26a-5p and let-7g-5p) overlapped between sperm and seminal plasma exosomes in HC versus CT pigs (Figure 5F). Interestingly, Spearman’s correlation coefficients of expression levels for overlapping DE miRNAs in CT and HC pigs were 0.994 and 0.894, respectively, which further emphasizes the close relationship between miRNAomes of sperm and seminal plasma exosomes and implies that testosterone deficiency may trigger fluctuations of this pattern. Prostasomes, one component of seminal plasma exosomes, are membrane-bound nanovesicles produced by acinar epithelial cells of the prostate gland. They are thought to play an important role in intercellular communication by transferring not only membrane components but also genetic material [55]. Recent studies suggested that prostasomes contain various bioactive molecules that improve semen quality [56,57] and enhance the fertilizing ability of spermatozoa in humans and boar [55,58]. Similar to prostasomes, epididymosomes are secreted by epididymal epithelial cells in an apocrine manner and play a major role in post-testicular sperm maturation by transferring proteins that are crucial for sperm to acquire fertilizing ability [59,60]. Furthermore, epididymosomes function as intercellular signaling mediators throughout the bovine epididymis by releasing distinct miRNA repertoires into the intraluminal fluid [61]. In this study, we found that prepubertal hemicastration induced expression pattern changes of sperm and seminal plasma exosome miRNAs in adult pigs and that their miRNAomes were significantly correlated. Taken together with previous research, it is reasonable to speculate that testosterone deficiencies induce changes in the miRNA components of seminal plasma exosomes secreted by the genital tract, which in turn transfer these changes to sperm cells. These appear to partially underlie the sperm miRNAome changes, which are responsible for the decline of sperm motility in hemicastrated pigs.

### 2.6. Validation of Small RNA-Seq Results Using Real-Time PCR

For validation purposes, we performed qRT-PCR for the expression of five overlapping DE miRNAs in CT and HC groups. The expression changes of miRNAs detected via qRT-PCR correlated with those in the small RNA-seq data (Spearman’s *r* = 0.939, *p* = 5.48 × 10^−5^), which highlighted the reliability of the small RNA-seq approach (Figure 5G).

## 3. Materials and Methods

### 3.1. Animal Materials and Experimental Treatments

The experimental procedures used in this study were approved by the Institutional Animal Care and Use Committee of Sichuan Agricultural University (Approval Number DKY-S20153307, 15 November 2015). A total of 30 Yorkshire boars from 15 pairs of full siblings were used in this study. At the age of seven days, one pig from each pair was randomly selected to be hemicastrated (HC) under anesthesia and another one remained intact as a normal control (CT). All boars in the hemicastration group (HC) were subjected to unilateral orchiectomy via a midline scrotal incision to remove the unilateral (left) testis, as described in a previous study [62]. Moreover, sham surgery was performed in the control group (CT) at the same time. All boars were weaned at the age of 25 days and transferred to a nursery and fed in the same pen. At the age of 60 days, each boar in the HC and CT groups was fed in an independent pen and all boars were fed a diet of the same nutritional level throughout this study until slaughter (at 10 months old) [63].

### 3.2. Measurement of Phenotypic Indexes

The phenotypic indexes (the body weight and circumference length) of all experimental boars were measured before slaughter and the weight of their main organs (i.e., heart, liver, spleen, lung, kidney, and right testis) was measured immediately after slaughter and further used for the calculation of organ indexes (organ index = organ weight/body weight × 100%).

### 3.3. Histomorphological Analysis

The right testis was taken immediately after death from each of the sacrificed pigs and cut in half along with the longitudinal axis using a sterile operating knife. Then one block sample (10 × 10 × 10 mm; ~1 g) of parenchymal tissue from the central portion was excised for immediate formalin fixation (10% buffered formalin solution) and was then paraffin-embedded for structural evaluation. Three micrometer thick skin sections were obtained at 150 μm intervals and were stained with hematoxylin-eosin. The mean diameter of a seminiferous tubule was calculated as the geometric average of the maximum and minimum diameter and 50 seminiferous tubules were measured for each sample in 10 randomly selected fields.

### 3.4. TUNEL Staining

The formalin-fixed, paraffin-embedded sections of testis from CT and HC pigs were analyzed using the Dead End^TM^ Fluorometric TUNEL System (Promega, Madison, WI, USA). The assay was done according to the manufacturer’s protocol. The fluorescein isothiocyanate (FITC) filter was used to find apoptotic cells, which showed up in green under a fluorescent microscope.

### 3.5. Isolation of Sperm and Seminal Plasma Exosome from the Fresh Semen

The ejaculates from each boar were collected using a manual collection method before slaughter [64] and only the spermatozoa-rich fractions of the ejaculates were collected and used in subsequent assays. Fresh semen was pre-diluted 1:1 with Beltsville Thawing Solution (BTS) and shipped on gel packs at about 17 °C to the laboratory. Diluted semen was placed in 50-mL Falcon tubes and centrifuged at 1000× *g* for 20 min at 4 °C to separate sperm and seminal plasma. Sperm pellets were washed twice using BTS at 1000× *g* for 20 min at 4 °C and incubated in somatic cell lysis buffer (0.1% SDS, 0.5% Triton X-100) on ice for 40 min to remove somatic cells. Next, the sperm was pelleted by centrifugation at 600× *g* for 5 min and then immediately put into liquid nitrogen until use. Seminal plasma exosomes were isolated by ultra-centrifugation and filtration according to a previously described method with some modifications [65]. Briefly, the seminal plasma was centrifuged at 16,000× *g* for 1 h at 4 °C to remove cell debris; next, the supernatant was successively filtered using 0.45 μm and 0.22 μm filters and then centrifuged at 120,000× *g* for 60 min at 4 °C. The pellets were washed by centrifugation (120,000× *g*, 60 min, 4 °C) twice using a wash buffer (30 mM TRIS, 130 mM NaCl, pH 7.6). Finally, the seminal plasma exosome pellet was then re-suspended in 250 μL nuclease free water and stored at −80 °C until use.

### 3.6. Sperm Apoptosis Detection by Flow Cytometry

An annexin V-FITC apoptosis detection kit was purchased from BD Pharmingen (San Diego, CA, USA) for the detection of sperm cell apoptosis by flow cytometry. A BTS fresh diluted semen sample (50 mL per pig) was centrifuged at 400× *g* for 12 min, before the supernatant was discarded. We washed the collected sperm cells with ethyleneglycol-bis-(β-amino-ethyl)-*N*,*N′*-tetraacetate (EGTA)/Hepes [66], collected sperm cells by centrifugation 400× *g* for 10 min, and discarded supernatant cells. Samples were subjected to double-staining of Annexin V-FITC/propidium iodide (PI) for 10 min and then proceeded to flow cytometry assays.

### 3.7. Atomic Force Microscopy (AFM)

To characterize the morphology of seminal plasma exosomes, samples was diluted 1:100 in deionized water, followed by 20 min fixation in freshly cleaved mica sheets. In order to provide a surface coated with formulations of a suitable density, the mica sheets were rinsed three times with deionized water and further dried with a filter paper before detection. Surface morphological characteristics of semen plasma exosomes were examined under an MFP-3D atomic force microscope (Asylum Research, Santa Barbara, CA, USA), as described in a previous report [65].

### 3.8. Western Blot Analysis

Total proteins were extracted from the sperm and the seminal plasma exosomes as previously described [65]. The protein concentrations were determined by a BCA Protein Assay Kit (Thermo Scientific, Rockford, IL, USA). Thirty milligrams of protein was separated on an 8% SDS-PAGE gel and then transferred to nitrocellulose membranes. The defatted milk was used to block the membranes for 2 h at room temperature and then the blocked membranes were incubated for 2 h with either a 1:1000 dilution of rabbit anti-alpha Tubulin (Abcam, Cambridge, MA, USA), a 1:1000 dilution of mouse anti-CD63 (Abcam), or a 1:200 dilution of mouse anti-TSG101 (Abcam). The washed blots were incubated for 90 min at room temperature with goat anti-mouse or anti-rabbit immunoglobulins (IgG) horseradish peroxidase (HRP) conjugated secondary anti-bodies (diluted 1:5000 in TBS containing 0.1% Tween-20 (TBST)). The antigen–antibody bands were scanned and visualized using a GS-700 imaging densitometer (Bio-Rad Laboratories, Hercules, CA, USA) and analyzed using Image Studio version 4.0 software (LI-COR Biosciences, Lincoln, NE, USA).

### 3.9. Sperm Quality Detection

Semen samples were preheated to 37 °C for 20 min and then 20 μL were dropwise added to a pre-warmed cell Counting-Chamber (Makler; 10 mm depth; Sefi Medical Instruments, Haifa, Israel). The sperm quality parameters were examined at 100× magnification with an Olympus BX41 microscope (Olympus Life Science Europe GMBH, Hamburg, Germany) equipped with phase-contrast optics and Sperm Class Analyzer 5 software (Microptic SL, Barcelona, Spain). A minimum of three replicates per seminal dose, with 1000 spermatozoa each, were analyzed. The motility parameters were evaluated in consecutive digitalized frames automatically acquired by Sperm Class Analyzer 5 software (Microptic SL, Barcelona, Spain) in each field. The definition of motility parameters, including VCL, VAP, VSL, ALH, LIN, STR, and WOB, was according to previous reports [67,68].

### 3.10. Small RNA Sequencing and Data Analysis

Total RNA from sperm and seminal plasma exosomes were extracted using Trizol Reagent (Takara, Shiga, Japan). Evaluation of the quality and integrity of extracted RNA was carried out on an Agilent 2100 Bioanalyzer (Agilent Technologies, Redwood City, CA, USA). Small RNA-seq libraries were constructed and then sequenced on an Illumina HiSeq 2500 sequencing platform ((Illumina, Santa Clara, CA, USA). In brief, small RNA fractions (length from 18–30 nucleotides) were isolated from extracted total RNA by polyacrylamide gel electrophoresis (PAGE, 15% Tris-borate-EDTA). The isolated small RNA fractions were ligated with 3′ and 5′ adaptors and reverse-transcribed to cDNA for PCR amplification. Finally, the PCR products were sequenced on Illumina HiSeq 2500 platform. The raw sequencing data from this study have been submitted to the NCBI Gene Expression Omnibus database (https://www.ncbi.nlm.nih.gov/geo) under the accession number GSE111985.

To identify the porcine miRNAs, the initial sequence was subjected to a series of stringent filters (such as removing low-quality reads, repeated sequences, and adaptor sequences). Filtered sequences were then mapped to a reference pig genome (Sscrofa11.1) with stringent criteria (0 mismatch in the first 18 bp) using Bowtie software [69]. Next, the retained sequences were mapped to the known porcine precursor miRNAs recorded in miRBase 21.0 (www.mirbase.org) for miRNA annotation and expression profiling (maximum of two mismatches allowed), using the NCBI Local BLAST.

### 3.11. Prediction and Functional Annotation of Sperm and Seminal Plasma Exosomes DE MiRNA Targets

To explore the potential functions of the sperm DE miRNAs, candidate miRNAs target prediction and functional enrichment analysis were conducted using DIANA-mirPath v3.0 online software (http://www.microrna.gr/miRPathv3) [32]. The predictions were made based on human mRNA–miRNA interactions using TarBase v7.0 (http://diana.imis.athena-innovation.gr/DianaTools/index.php?r=tarbase/index), as porcine miRNAs were not available in the current version of the abovementioned algorithm. The gene ontology (GO) terms (including biological process (BP), cellular component (CC) and molecular function (MF)) and Kyoto Encyclopedia of Genes and Genomes (KEGG) pathways were selected for the functional annotation of predicted target genes.

### 3.12. miRNA Transfection in Sperm

On the basis of the small RNA-seq results and bioinformatics prediction, miR-26a-5p and let-7g-5p were selected to further verify their biological functions. The specific miRNA mimics and inhibitors were purchased from RIBOBIO (Guangzhou, China). Following the instructions in the user manual, sperm transfection was carried out using X-treme GENE siRNA Transfection Reagent (Roche, Basel, Switzerland). Briefly, the swim-up methods were used for preprocessing the diluent semen to obtain the portion containing high-quality sperm [57]. Separated sperm was washed twice using BTS at 1000× *g* for 20 min. Next, five groups of sperm were prepared, including a control (no transfection); mimic-transfected; mimic control-transfected; inhibitor-transfected; and inhibitor control-transfected. The miRNA transfection solution and sperm medium were sufficiently mixed at a final concentration of 50 nM for the mimic/mimic control-transfected groups, or 100 nM for the inhibitor/inhibitor control-transfected groups. All groups were placed at 17 °C for 8 h and then used for the following experiment.

### 3.13. Dual Luciferase Reporter Assay

The potential binding sites of miR-26a-5p and let-7g-5p in porcine *PTEN* and *PMAIP1* mRNA sequences were predicted by TargetScan [40] and RNAhybrid [41], respectively. Luciferase activity assays were performed to evaluate a relationship between candidate miRNA and its potential target genes. In brief, the potentially targeted mRNAs (*PTEN* and *PMAIP1*) containing binding sites of miR-26a-5p and let-7g-5p, respectively, were synthesized by Tsingke (Chengdu, China) (Figure 4A). The sequences were cloned into the 3′ end of the firefly (luc2) luciferase reporter gene of the pmirGLO plasmid (Promega, Madison, WI, USA). Porcine kidney cells line (PK15) were cultured in 24-well plates and when the cells reached about 70% confluence, the recombinant pmirGLO vector was co-transfected with ssc-miR-26a-5p/ssc-let-7g-5p mimics or negative control oligos into these cells by Lipofectamine 3000 (Invitrogen, Carlsbad, CA, USA). PK15 cells were collected 48 h after the transfection and dual-luciferase activity was measured using the Dual-Luciferase Reporter Assay System kit (Promega), according to the manufacturer’s instructions.

### 3.14. qRT–PCR

Total RNA was isolated from the sperm and the seminal plasma exosomes using TRIzol Reagent (Invitrogen), following the instructions in the user manual. The reverse transcription of mRNA and miRNA were carried out by PrimeScript RT reagent Kit (Takara, Dalian, China) with gDNA Eraser and Mir-X™ miRNA First Strand Synthesis Kit (Takara), respectively. The qRT-PCR was carried out on a CFX96 detection system (Bio-Rad Laboratories) with a SYBR Premix Ex Taq kit (Takara). All reactions were performed in triplicate and the thermal cycling parameters were as follows: 5 min at 95 °C, followed by 40 cycles of three-step amplification (15 s at 95 °C, 15 s at 60 °C, and 30 s at 72 °C). Relative expression levels of mRNAs and miRNAs were normalized by the expression of *GAPDH* and *U6*, respectively, and were calculated using the comparative 2-^ΔΔCt^ method [70]. Primer sequences used in this study are shown in Table S5. All primers used in this study were synthesized by Tsingke (Chengdu, China).

### 3.15. Statistical Analysis

For small RNA-seq data, we used the freely available R software [71] to analyze the difference in miRNAs expression. Statistical significance was calculated using a paired, two-tailed *t*-test. The computed *p*-values for the *t*-test were adjusted with the use of Benjamini–Hochberg false discovery rate (FDR) [72] correction. Differentially expressed miRNAs were defined as those exhibiting a Benjamini-corrected *p*-value ≤ 0.05 and a fold-change >2 or <0.5 in HC versus CT.

For other data, one way ANOVA with Tukey’s *post hoc* test and Student’s *t*-test were employed to evaluate the statistical significance for comparisons of multiple groups and two groups, respectively, using SPSS 19.0 software (SPSS Inc., Chicago, IL, USA).

## 4. Conclusions

In this study, we carried out hemicastration in prepubertal Yorkshire boars and successfully established a testosterone deficiency model, which showed obvious compensatory hyperplasia in the remaining testis and a significant enhancement of sperm apoptosis accompanied by a dramatic decline in sperm motility. Using the small RNA-seq approach, we compared miRNA profiles of sperm cells between HC and CT boars and revealed that DE miRNAs were mainly enriched in pathways involved in cell apoptosis, including mTOR and p53 signaling pathways. Furthermore, gain- and loss-of-function analyses and dual luciferase assays demonstrated that endogenous miR-26a-5p and let-7g-5p have potential anti-apoptotic and pro-survival functions in sperm cells by targeting *PTEN* and *PMAIP1* genes. Further comparisons revealed similarities in miRNA profiles between seminal plasma exosomes and sperm cells, both in HC and CT groups. Our study revealed that miRNAs in sperm cells and seminal plasma exosomes have important functions in the male reproductive system, which could be exploited for future treatments or novel diagnostic markers for male infertility.

## Supplementary Materials

Supplementary materials can be found at http://www.mdpi.com/1422-0067/19/4/1233/s1.

## Abbreviations

miRNA	microRNA
MAPK	mitogen-activated protein kinase
mTOR	mammalian target of rapamycin
PMAIP1	phorbol-12-myristate-13-acetate-induced protein 1
*PTEN*	phosphatase and tensin homologue
tRF	tRNA-derived RNA fragment
VSL	straight line velocity
VCL	curvilinear velocity
VAP	average path velocity
ALH	amplitude of lateral head displacement
WOB	wobble
LIN	linearity
STR	straightness
qRT-PCR	quantitative real-time PCR
