# Supplementary material for: Testosterone-Dependent miR-26a-5p and let-7g-5p Act as Signaling Mediators to Regulate Sperm Apoptosis via Targeting PTEN and PMAIP1
Source: Int J Mol Sci. 2018 Apr 18;19(4):1233. doi: 10.3390/ijms19041233 (PMC5979296; doi:10.3390/ijms19041233)
Supplement: Supplementary file 1 [file ijms-19-01233-s001.zip › Figure S1.pdf]

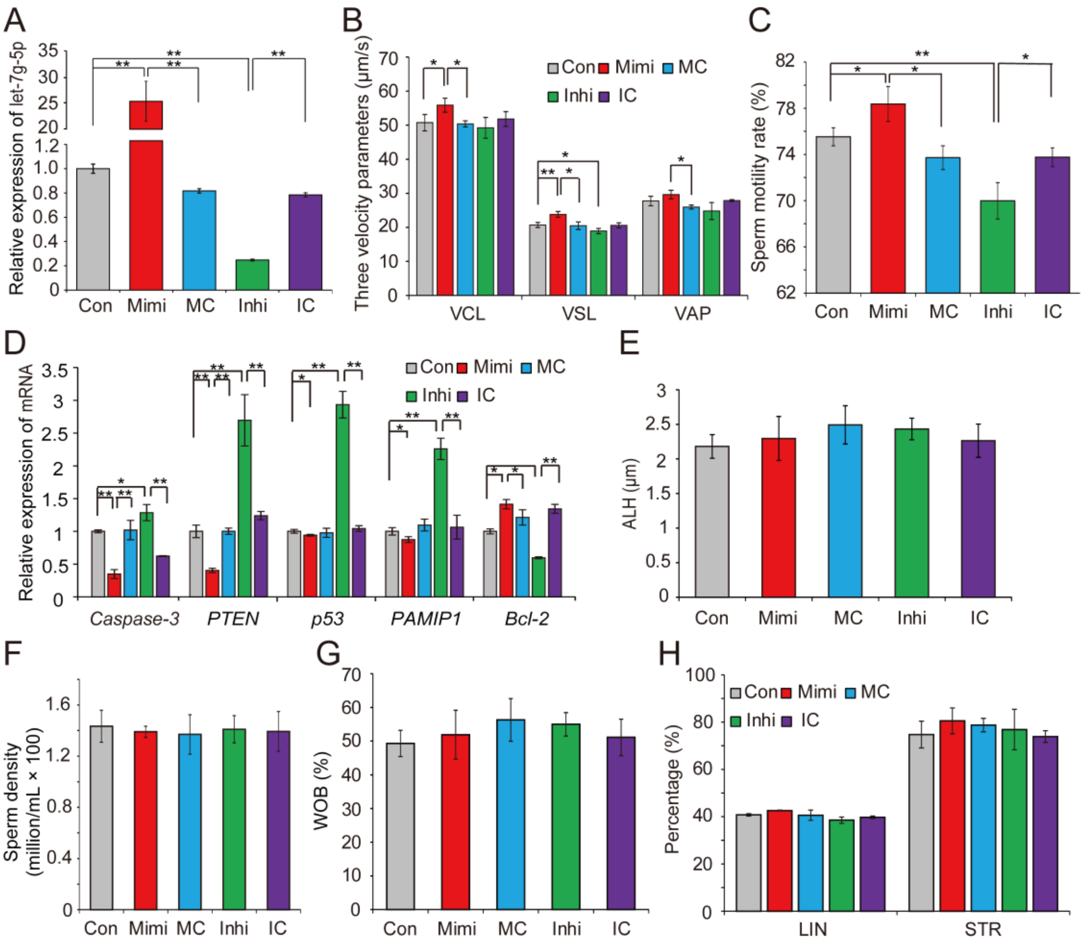

Figure S1. The let-7g-5p-mediated regulation of sperm quality. (**A**) Relative expression levels of let-7g-5p in control, mimic-, and inhibitor-transfected sperm. (**B**) Effect of let-7g-5p mimic and inhibitor on three velocity parameters of sperm cells, including curvilinear velocity (VCL, μm/s), average path velocity (VAP, μm/s), and straight line velocity (VSL, μm/s). (**C**) Effect of let-7g-5p mimics and inhibitor on sperm motility rate (%). (**D**) Effect of let-7g-5p mimics and inhibitor on the expression levels of apoptosis-related genes. No significant changes were observed in (**E**) amplitude of lateral head displacement (ALH, μm), (**F**) sperm density (million/mL), (**G**) motility parameter wobble (WOB, %), or (**H**) linearity (LIN, %) and STR (%).Con, control; Mimi, mimics; MC, mimic control; Inhi, inhibitor; IC, inhibitor control. CT and HC represent the prepubertally hemicastrated Yorkshire boars and normal controls, respectively. Three independent experiments were performed in triplicate and all data are expressed as means ± SD. * $p < 0.05$, ** $p < 0.01$.
