# Supplementary material for: Testosterone-Dependent miR-26a-5p and let-7g-5p Act as Signaling Mediators to Regulate Sperm Apoptosis via Targeting PTEN and PMAIP1
Source: Int J Mol Sci. 2018 Apr 18;19(4):1233. doi: 10.3390/ijms19041233 (PMC5979296; doi:10.3390/ijms19041233)
Supplement: Supplementary file 1 [file ijms-19-01233-s001.zip › Table S5 List of gene-specific primers for qRT-PCR.doc]

**Table S5.** List of gene-specific primers used for qRT-PCR.

| **Gene Name** | **Sequence (5' to 3')** |
| --- | --- |
| *Caspase-3* | F- TGGTACAGATGTGGATGCT  R- TGGGTTTGCCAGTTAGAGT |
| *PTEN* | F- TGGAAAGGGACGAACTGGTG  R- TCGTGTGGGTCCTGAATTGG |
| *P53* | F- CCAGTGCAAAAGAAGAAGCCA  R- CTCGGAACATCTCGAAGCGT |
| *Bcl-2* | F- GGCAACCCATCCTGGCACCT  R- AACTCATCGCCCGCCTCCCT |
| *PMAIP1* | F- AAGGAGGTCTCGTAGGAACAC  R- GAACTGAATGGCACACTCG |
| miR-26a-5p | F- TTCAAGTAATCCAGGATAGGCT |
| Let-7g-5p | F- TGAGGTAGTAGTTTGTACAGTT |
| miR-182-5p | F- TTTGGCAATGGTAGAACTCACACT |
| Let-7i-5p | F- TGAGGTAGTAGTTTGTGCT |
| miR-30b-5p | F- TGTAAACATCCTACACTCAGCT |
| *GAPDH* | F- AATGACCCCTTCATTGACCTC  R- CCTTGACTGTGCCGTGGAACT |
| *U6* | F- TTATGGGTCCTAGCCTGAC  R- CACTATTGCGGGTCTGC |

Note: *GAPDH* and *U6* were used as housekeeping genes for normalising mRNA and miRNA, respectively.
